# Trends in and correlations between antibiotic consumption and resistance of *Staphylococcus aureus* at a tertiary hospital in China before and after introduction of an antimicrobial stewardship programme

**DOI:** 10.1017/S0950268818003059

**Published:** 2018-11-16

**Authors:** Di Zhang, Kai Cui, Taotao Wang, Haiyan Dong, Weiyi Feng, Chen Ma, Yalin Dong

**Affiliations:** 1 Department of Pharmacy, The First Affiliated Hospital of Xi'an Jiaotong University, Xi'an 710061, China; 2 Department of Management of the Economy, Xi'an University of Posts and Telecommunications, Xi'an 710061, China; 3 Department of Clinical Laboratory, The First Affiliated Hospital of Xi'an Jiaotong University, Xi'an 710061, China

**Keywords:** Antibiotic consumption, antibiotic resistance, antimicrobial stewardship, MRSA, *Staphylococcus aureus*

## Abstract

The overuse of antibiotics and the rapid emergence of antibiotic resistance prompted the launch of an antimicrobial stewardship programme in 2011. This study aimed to investigate the trends and correlations between antibiotic consumption and resistance of *Staphylococcus aureus* in a tertiary hospital of northwest China from 2010 to 2016. Trends were analysed by linear regression, and correlations were assessed by an autoregressive integrated moving average model. The total consumption of antibiotics halved during the 7-year study period, while the rates of resistance of *S. aureus* decreased significantly or remained stable; methicillin-resistant *S. aureus* (MRSA) declined markedly, from 73.3% at the beginning of the study to 41.4% by the end. This latter decrease was significantly correlated with the consumption of several classes of antibiotics. In conclusion, reduction in antibiotic use impacted significantly on resistance rates and contributed to a decline in MRSA prevalence.

## Introduction

Antibiotic resistance is a serious threat to public health [1]. Infections caused by resistant bacteria are difficult to treat, leading to high mortality and morbidity, prolonged hospital stays and excessive costs [2]. The overuse of antibiotics is a crucial contributory factor to the rapid emergence of resistant microorganisms. To counteract this, guidelines for the control and application of antibiotics have been implemented in China, first in 2004 [3] and then in 2009 [4]. However, a report in 2009 highlighted the continual use of antibiotics in China particularly in seasonal influenza cases (75%) and hospital inpatients (80%) [5].

In response to a WHO programme in 2011 [6] to stem the global tide of antibiotic resistance, a 3-year rectification scheme on the appropriate use of antibiotics was launched by the health authorities in China [7]. The policy aimed to strengthen the management of antibiotics in clinical applications by setting targets for restricting the types of antibiotics and their usage in Chinese hospitals and was subsequently enforced by law in 2012 [8].

In July 2011, several antimicrobial control regulations were introduced in the First Affiliated Hospital of Xi'an Jiaotong University (FAHXJU). This study reports the trends in, and correlations between, antibiotic consumption and resistance of *Staphylococcus aureus*, and particularly methicillin-resistant strains (MRSA), in this hospital over the period 2010–2016.

## Methods

### Design and setting

FAHXJU is a 2541-bed tertiary-care teaching hospital located in the northwest of China. Antibiotic susceptibility patterns of inpatient isolates were monitored by the Clinical Microbiology Laboratory and antibiotic usage was obtained from the computerised database of the Department of Pharmacy. The data were collected per quarter from 2010 to 2016. The need for an informed consent was waived by the ethics review board.

### Antibacterial stewardship

In brief, the national rectification scheme introduced in April 2011 [7], restricted tertiary care hospitals to 50 antimicrobials with reduction targets of 60% and 20% of all prescriptions for hospitalised patients and outpatients, respectively. Consumption of antibiotics was expressed as the antibiotic use density [9], with total consumption limited to ⩽400 defined daily doses (DDDs) per 1000 patient-days (PDs). The prophylactic use of antibiotics in clean operations was specified to be used for <30% of patients and not given for more than 24 h. Antibiotics were classed as non-restricted, restricted or for specialist use. Resident physicians were only allowed to prescribe non-restricted antibiotics, attending physicians both non-restricted and restricted antibiotics, and specialist agents such as carbapenems, glycopeptides, linezolid and aztreonam, could only be prescribed by associate chief of chief physicians with specialised knowledge. These protocols were implemented from July 2011.

### Microbiology data

Identification and susceptibility testing of bacterial isolates were performed using the automated VITEK 2 automated system (bioMérieux, Lyons, France). All *S. aureus*-positive clinical specimens from inpatients were included in the database. The isolates were classed as susceptible, intermediate susceptible or resistant, according to Clinical and Laboratory Standards Institute criteria [10]. Duplicate isolates were excluded.

### Statistical analysis

The trends in antibiotic resistance, antibiotic consumption and medical quality indicator data were analysed by linear regression with SPSS version 19.0. Correlations between antibiotic consumption and antibiotic resistance were analysed by autoregressive integrated moving average (ARIMA) models, allowing for possible lag times of a half year [11, 12]. We fitted an ARIMA model according to Box–Jenkins methodology and performed the following steps with STATA version 15.0. Firstly, we checked whether the series needed to be differentiated with the augmented Dickey–Fuller test and then created the model by determining the values of the remaining parameters ‘*p*’ and ‘*q*’ of the ARIMA (*p*, *d*, *q*) model with autocorrelation and partial autocorrelation. Subsequently, model parameters were estimated by the Maximum Likelihood method and finally checked for adequacy of the model. Independent variables with *P* < 0.05 in the univariate ARIMA models were included in multivariate ARIMA regression model. All reported *P* values were two-sided, and *P* < 0.05 were considered statistically significant.

## Results

### Medical quality indicators

Administrative data were obtained regarding the number of hospitalisations, hospital stays and other medical quality indicators over the study period (Table 1). The number of hospitalisations increased significantly by year (*β* = 8.411, *P* < 0.05) and values increased by 74.19%. The average number of hospital stays decreased significantly (*β* = −0.637, *P* < 0.05) by 30.6%, as did the mortality rate (*β* = −0.083, *P* < 0.05). Likewise, significant reductions were also recorded for the incidence of nosocomial infections (*β* = −0.443, *P* = 0.001), and antibiotic usage (*β* = −5.570, *P* = 0.026).

**Table 1.** Annual medical quality indicators in the First Affiliated Hospital of Xi'an Jiaotong University, 2010–2016

Medical quality indicators	2010	2011	2012	2013	2014	2015	2016	Trend	Slope (*β*)	*P*
Number of hospitalisations (1000 patients)	68.15	76.25	89.06	96.78	103.40	110.99	118.71	Increasing	8.411	0.000
Average of hospital stays (days)	12.07	11.66	10.64	9.53	9.34	8.93	8.38	Decreasing	−0.637	0.000
Mortality rate (%)	0.86	0.82	0.66	0.52	0.49	0.42	0.41	Decreasing	−0.083	0.000
Nosocomial infection rate (%)	2.99	3.34	2.49	1.56	1.2	0.93	0.89	Decreasing	−0.443	0.001
Antibiotics usage rate (%)	63.10	45.80	25.56	28.27	26.95	25.16	24.41	Decreasing	−5.570	0.026

### Trends in *S. aureus* resistance and consumption of selected antibiotics

**Fig. 1.**
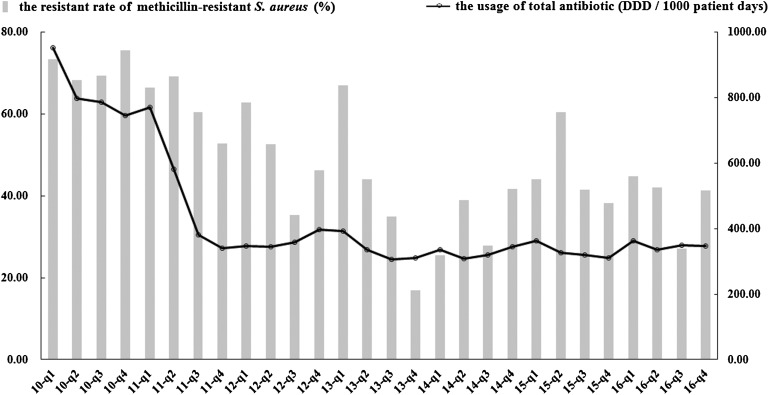
Trends in the rate of resistance to methicillin-resistant *S. aureus* (MRSA) and the quarterly usage of total antibiotics during 2010–2016. The usage of total antibiotics is represented as DDD per 1000 patient days on the right *y*-axis. Resistance rates are shown on the left *y*-axis. DDD, defined daily dose.

In total, 3975 *S. aureus* isolates were collected: 447 isolates in 2010, 732 in 2011, 728 in 2012, 582 in 2013, 382 in 2014, 532 in 2015 and 572 in 2016. Table 2 shows the trends of antibiotic resistance of these isolates over the 7-year period. Resistance rates for all 12 selected antimicrobials decreased significantly or remained stable; concomitantly the incidence of MRSA declined markedly, from 73.3% in quarter 1 of 2010 to 41.4% in quarter 4 of 2016 (*P* < 0.05, Fig. 1). Both vancomycin and linezolid remained active against all strains of *S. aureus.* Significant decreases in the consumption of oxacillin, benzylpenicillin, erythromycin, ciprofloxacin, levofloxacin and gentamicin were also noted in contrast to increases in consumption of clindamycin, moxifloxacin, vancomycin and sulfamethoxazole/trimethoprim (*P* < 0.05). Less substantial changes in the consumption of linezolid were evident. Tetracyclines were not available for prescription in the hospital during the study period.

**Table 2.** Trends in and correlation between the rates of resistance to *S. aureus* and the selected antibiotics in consumption during 2010–2016

Antibiotics		Resistance^a^			Consumption^b^			Correlation				
Selected	Levels	Average^c^	Trend	*P*	Average^c^	Trend	*P*	Lag (quarter)	Coefficient	s.e.	*T* statistic	*P*
Oxacillin	A	44.45% (38.45–65.50%)	Decreasing	0.000	0.00 (0.00–0.06)	Decreasing	0.000	0	0.334	0.122	2.74	0.006
Benzylpenicillin	A	96.45% (94.63–98.08%)	Stable	0.237	2.16 (1.33–3.80)	Decreasing	0.000	0	−0.005	0.002	−0.22	0.026
Erythromycin	A	73.65% (66.55–79.77%)	Decreasing	0.000	0.79 (0.52–1.31)	Decreasing	0.000	/	0.005	0.009	0.52	0.603
Clindamycin	A	66.55% (38.98–78.26%)	Decreasing	0.000	1.55 (0.67–2.03)	Increasing	0.000	1	0.065	0.031	2.09	0.036
Ciprofloxacin	A	43.03% (28.93–60.08%)	Decreasing	0.000	2.10 (1.90–2.87)	Decreasing	0.000	2	0.085	0.029	2.91	0.004
Levofloxacin	A	41.05% (22.23–56.95%)	Decreasing	0.000	16.44 (14.42–18.68)	Decreasing	0.010	/	0.003	0.005	0.64	0.519
Moxifloxacin	B	31.45% (18.48–43.45%)	Stable	0.108	23.37 (19.18–28.04)	Increasing	0.000	1	0.017	0.007	2.49	0.013
Gentamicin	A	53.54% (32.75–66.00%)	Decreasing	0.000	20.84 (16.11–24.00)	Decreasing	0.000	0	0.017	0.006	2.87	0.004
Sulfamethoxazole and trimethoprim	A	23.25% (18.87–28.88%)	Stable	0.730	5.24 (4.07–6.61)	Increasing	0.000	2	0.17	0.007	2.42	0.016
Tetracycline	B	52.03% (39.20–61.18%)	Decreasing	0.000	/	/	/	/	/	/	/	/
Vancomycin	C	/	/	/	3.07 (1.96–3.83)	Increasing	0.000	/	/	/	/	/
Linezolid	C	/	/	/	2.49 (1.54–2.88)	Stable	0.560	/	/	/	/	/

A, non-restricted; B, restricted; C, specialist antibiotic.

a Resistance is shown as the percentage of resistant isolates per quarter.

b Antibacterial consumption is shown as defined daily dose/1000 inpatient-days per quarter.

c The average values are presented as median (interquartile range).

Positive correlations were found for resistance to the agent and the consumption of oxacillin and gentamicin in lag quarter 0, with coefficients of 0.334 and 0.017 (*P* < 0.05), respectively (Table 2). Other significant (*P* < 0.05) correlations of resistance and consumption were noted for clindamycin and moxifloxacin in lag quarter 1, and for ciprofloxacin and sulfamethoxazole/trimethoprim after 6 months (lag quarter 2). The only negative correlation was for benzylpenicillin in lag quarter 0 (*P* = 0.026).

### Incidence of MRSA and antibiotic consumption

The total consumption of antibiotics decreased from 951.88 DDDs/1000 PDs in quarter 1 of 2010 to 346.45 DDDs/1000 PDs in quarter 4 of 2016 (*β* = −0.653, *P* < 0.05, Fig. 1). This decrease was statistically significant for several antibiotic classes notably the cephalosporins, monobactams, aminoglycosides, imidazole derivatives, steroid antibacterials, macrolides, lincosamides and streptogramins. However, significant increases in consumption were noted for carbapenems, glycopeptides and sulphonamides (Table 3). The penicillins and quinolones remained stable over the study (*P* > 0.05).

**Table 3.** Trend in the consumption of antibiotic classes during 2010–2016

Antibiotic		Antibiotic consumption^a^			
Classes	Levels	Average	Trend	Slope (*β*)	*P*
Penicillins and enzyme inhibitor	Non-restricted; restricted	29.41 (23.82–35.05)	Stable	−0.012	0.185
Cephalosporins	Non-restricted; restricted; specialist	168.74 (149.74–213.13)	Decreasing	−0.282	0.000
Carbapenems	Specialist	24.54 (20.08–37.44)	Increasing	0.043	0.000
Monobactams	Specialist	0.59 (0.22–3.84)	Decreasing	−0.013	0.000
Glycopeptides	Specialist	5.79 (4.74–7.71)	Increasing	0.007	0.000
Oxazolidinone	Specialist	2.49 (1.54–2.88)	Stable	0.001	0.560
Macrolides, lincosamides, and streptogramins	Non-restricted; restricted	13.42 (10.89–32.71)	Decreasing	−0.259	0.000
Quinolones	Non-restricted; restricted	46.42 (39.44–52.17)	Stable	−0.020	0.096
Aminoglycosides	Non-restricted; restricted	27.93 (21.17–37.10)	Decreasing	−0.053	0.000
Imidazole derivatives	Non-restricted	23.75 (20.48–29.44)	Decreasing	−0.069	0.000
Steroid antibacterials	Non-restricted	0 (0–0.03)	Decreasing	−0.001	0.000
Sulphonamides	Non-restricted	5.24 (4.07–6.61)	Increasing	0.007	0.000
Total	/	346.44 (329.15–396.49)	Decreasing	−0.653	0.000

a Antibacterial consumption is shown as defined daily dose/1000 inpatient-days per quarter, and the average values are presented as median (interquartile range).

In the univariate ARIMA models, the use of nine classes of antibiotics namely cephalosporins, monobactams, glycopeptides, oxazolidinone, quinolones, aminoglycosides, imidazole derivatives, steroid antibacterials and sulphonamides were associated with the MRSA isolation rate (*P* < 0.05). Meanwhile, the isolation rate of MRSA would rise with increased usage of the total antibiotics in the next quarter (lag 1, *P* < 0.05). However, in the multivariate ARIMA model (Table 4), the MRSA rate was expected to mirror the increased usage of imidazole derivatives, sulphonamides and monobactams. Nevertheless, negative correlations were found between the rise in MRSA and the usage of glycopeptides, oxazolidinone and aminoglycosides.

**Table 4.** Multivariate ARIMA (0, 1, 1) model of the antibiotic classes in consumption associated with the prevalence of resistance to MRSA during 2010–2016

Independent variables^a^	Lag (quarter)	Coefficient	s.d.	*T* statistic	*P*
Monobactams	2	0.023	0.006	3.80	0.000
Glycopeptides	0	−0.030	0.011	−2.83	0.005
Oxazolidinone	2	−0.063	0.012	−5.38	0.000
Aminoglycosides	2	−0.009	0.003	−3.06	0.002
Imidazole derivatives	1	0.008	0.001	6.41	0.000
Sulphonamides	1	0.044	0.011	4.01	0.000
Autoregressive term^b^	1	−0.010	2.05 × 10^−8^	−4.90 × 10^5^	0.000

ARIMA, autoregressive integrated moving average; MRSA, methicillin-resistant *S. aureus*.

a The antibiotic classes in consumption mean as defined daily dose/1000 inpatient-days per quarter.

b The autoregressive term represents the past value of the resistance.

## Discussion

In this study, we tracked the volume of antibiotic consumption over a 7-year period to gain insights into trends and correlations between antibiotic consumption and resistance of *S. aureus* in our university hospital. The first noteworthy finding was that antibiotic usage reduced significantly over the study period with total consumption falling by more than half. These changes are likely attributable to the implementation of the antimicrobial stewardship programme, and similarly significant reductions have been noted at other hospitals in China following this initiative [13, 14]. Nationally, total antibiotic usage in hospitals decreased from 67.3% to 36.8% in hospitalised patients, while consumption fell from 776 DDDs/1000 PDs in 2010 to 457 DDDs/1000 PDs in 2017 [14]. However, use of the ‘specialist’ glycopeptides and carbapenems increased significantly during the study period. Nevertheless, serious overconsumption of carbapenems remained after implementation of the stewardship programme and this prompted the national health authorities to issue a further notice on the rational use of carbapenems and other specialist antibiotics [15].

The second noteworthy finding is that there was a significant reduction in the rate of resistance of *S. aureus* to antimicrobials, none of which showed an upward trend; the rate of MRSA infections also declined significantly. According to national antimicrobial resistance surveillance reports [16, 17], the prevalence of MRSA declined modestly from 2014 (36%) to 2016 (34.4%), with earlier data not being available. In our hospital, the MRSA rate began to decrease from quarter 2 of 2011 (Fig. 1) and at the end of the study stood at 41.4% but was still higher than the national average. Most importantly, all MRSA isolates remained susceptible to vancomycin and linezolid throughout the study. The declining prevalence of resistant *S. aureus*, particularly MRSA, might be partly explained by the overall reduction in antibiotic use as a consequence of the antimicrobial stewardship programme.

While antibiotic use is a key risk factor for the emergence of bacterial resistance, their relationship is complex [18]. The correlation coefficients showed that the rate of resistance to benzylpenicillin decreased immediately by 0.005 after increased use of this agent and likewise resistance to oxacillin increased immediately by 0.334 after an increase in its use. However, it should be noted that oxacillin was not prescribed in our hospital after July 2011, and therefore this anomaly requires further investigation.

The multivariate ARIMA models also revealed positive correlations between the prevalence of MRSA and the usage of imidazole derivatives, sulphonamides and monobactams, but negative correlations between MRSA prevalence and usage of glycopeptides, oxazolidinone and aminoglycosides. This finding is contrary to a meta-analysis which concluded that the prevalence of MRSA was associated with the usage of glycopeptides, extended-spectrum cephalosporins and fluoroquinolones [19]. Likewise, Lai *et al*. [20] reported a significant correlation between the increased usage of extended-spectrum cephalosporins, *β*-lactam/*β*-lactamase inhibitor combinations and carbapenems, and a decreased prevalence of MRSA. These inconsistencies might be due to differences in strain characteristics and antibiotic-prescribing practices between different countries [11, 21]. Also, it is noteworthy that correlations revealed by regression analyses may not necessarily be causal relationships [18]. For example, our model suggested that the prevalence of MRSA would decrease immediately by 0.030 on an increase in glycopeptide use and by 0.063 after 6 months following increased use of oxazolidinone, which are two main classes of antibiotics used to treat MRSA. By contrast, Tacconelli *et al*. [19] showed in a meta-analysis a positive correlation between the rate of MRSA and the glycopeptide usage. However, this was not corroborated in an eight-country study, with more than 5% decrease of MRSA infection, which found only a negative correlation between glycopeptide use and MRSA rates [22]. Moreover, Mascitti *et al*. [23] suggested that prior vancomycin use and reduced vancomycin susceptibility was a significant risk factor for patients with methicillin-susceptible *S. aureus* infection, but not for those with MRSA infection. It is more logical that the increasing usage of glycopeptides in some patients might decrease MRSA prevalence, but accompanied by a degree of reduced susceptibility to this class of antibiotic. The available evidence does not support the view that increasing the use of some antibiotics may decrease the prevalence of MRSA, and further research is needed to explore this concept in areas with a high prevalence of MRSA.

The finding of a positive correlation between the MRSA rate and consumption of imidazole derivatives (or sulphonamides) is also noteworthy. However, these agents are not normally used for the treatment of *S. aureus* infections and are not restricted in use under the stewardship programme; they are generally inexpensive and are thought to have little effect on antibiotic resistance. Some studies have reported exposure to agents with anti-anaerobic activity as a risk factor for candidemia, particularly for *Candida glabrata* bloodstream infection [24, 25]. In the natural environment, bacteria exist in close association with other microorganisms and this facilitates their survival [26]. Our findings indicate that all antibiotics should be used with caution irrespective of their degree of restriction of use.

This study was subject to several limitations. First, it had a single-centre, retrospective, observational design, and used aggregate data with an inherent risk of bias, since data from individual patients might give different results. Secondly, the density of antibiotic use might not accurately reflect the real antibiotic use in adults with renal impairment; however, it is the most commonly applied metric for measuring antibiotic use. Thirdly, we were unable to differentiate infection from colonisation with *S. aureus*. Nevertheless, we believe that this study based on longitudinal data obtained over several years makes a meaningful contribution to our understanding of the relationship between usage of antimicrobials and resistance rates as a consequence of the implementation of the antimicrobial stewardship programme in our tertiary care hospital. Since the end of the study, resistance rates of *S. aureus* have remained significantly reduced, with a continuous decline in MRSA infections. The mechanisms underlying antibiotic consumption and MRSA resistance are worthy of further study.
